# Mechanical Measurements of Cells Using AFM: 3D or 2D Physics?

**DOI:** 10.3389/fbioe.2020.605153

**Published:** 2020-11-19

**Authors:** Yifat Brill-Karniely

**Affiliations:** Faculty of Medicine, The School of Pharmacy, Institute for Drug Research, The Hebrew University of Jerusalem, Jerusalem, Israel

**Keywords:** atomic force microscope (AFM), cell mechanics, membrane elasticity, area expansion modulus, contact mechanics, Young's modulus, dimensionality, physical modeling

## Introduction

The mechanical properties of biological samples are tightly linked to normal and pathologic functions. For example, there is correlation between cancer aggressiveness and the mechanical deformability of tumor cells, leading to highly potential clinical applications (Quan and Kim, 2016; Brill-Karniely et al., 2020; Kozminsky and Sohn, 2020). Atomic force microscopy (AFM) is a commonly used method for high-resolution mechanical measurements. The AFM experiments are based on detecting the response of a sample to force exerted by an indenting probe. Analysis models are then used for quantification of cell properties such as the Young's modulus.

The default AFM analysis models are three-dimensional (3D), primarily contact mechanics theories that assume large thickness of the sample relative to the indentation. However, under certain conditions, the AFM load can cause deformation of the cell membrane with negligible effect on other cell components. Since membrane thickness is of a few nanometers, and indentation depths are typically much larger, a question is raised about the validity of 3D models in those cases. The dimensionality in AFM mechanical cell measurements has not been previously addressed to the best of our knowledge. We claim here that indentations that perturb only the cell membrane need to be analyzed by two-dimensional (2D) theories, whereas 3D models, such as contact mechanics theories, cannot represent those cases. Specifically, when using 3D models in analyzing indentations with sharp tips whose apex is of a few nanometers, shallow depths up to ~200 nm should not be included in the fitting range. On the other hand, we demonstrate that using 2D models, shallow (~100 nm) indentations of sharp tips can provide instructive information about membrane tension moduli.

## AFM Mechanical Measurements of Biological Samples

In AFM force spectroscopy, curves of the force against the indentation depth provide mechanical insight about the sample (Kilpatrick et al., 2015; Gavara, 2017). The approach part of the curves provides information on the sample elasticity. The derivative of the curve is affected by the resistance of the sample to the applied force—the lower is the slope, the higher is the elasticity. The elasticity parameter obtained from such analysis is often regarded as the Young's modulus, suggesting that it can represent the local rigidity of the sample. This derivation is valid when the sample behaves in an elastic manner. In the case of cells, it can be a reasonable assumption provided that the experimental conditions are properly tuned. The elastic moduli are obtained by fitting the force–indentation plots to appropriate physical models. The theories used for cell measurements analysis are based on 3D physics. Contact mechanics theories, which are the default models of AFM analysis software, are the most common (Kilpatrick et al., 2015; Gavara, 2017). Occasionally, other schemes such as finite element models are applied (Lulevich et al., 2010; Zhang and Zhang, 2011; Vahabikashi et al., 2019). Mechanical indentation of samples can be made either with tips of a-few-nanometer apexes, or with larger particles, usually microbeads. The probes, and mainly the sharp tips, often vary in their shape. Common geometries include conical, spherical, pyramidal, and parabolic tips (Kilpatrick et al., 2015; Gavara, 2017). Specific contact mechanics models account for different probe geometries as well as for aspects such as the presence of attractive interactions and their range. Under simplifying approximations different versions of contact mechanics provide a power law of the force–indentation (*F*-δ) curves:

(1) $$\begin{array}{l} F\left(\delta \right)=C\left(E,R\right)\cdot \delta^{\alpha } \end{array}$$

The prefactor *C*(*E, R*) depends on the cell's Young's modulus, *E*, and can depend on the dimensions of the probe, generally termed here as *R*. It also depends on the Poisson's ratio, which describes the transversal expansion of the sample due to the load; however, this property is usually taken as a fixed value of 0.5, similar to incompressible rubber. The power exponent, α, as well as *C*(*E, R*) vary with the details of the probe geometry.

## The “Elastic Half-Space” Assumption

All of the contact mechanics theories—by definition—are based on the assumption that the cell is an “elastic half-space,” namely a semi-infinite elastic body bounded by a plane surface (Johnson, 1985). In other words, the cell is assumed to be elastic (tends to return its original geometry after application of force) and with dimensions that are much larger than the indentation.

The conditions underlying the assumption of cell elasticity have been discussed in detail, referring to the need for careful adjustments of experimental definitions like the indentation depth and fit range, the cantilever stiffness, the locations of the pressing points, and the extension/retraction speed (Kilpatrick et al., 2015). However, the “half-space” requirement of large sample dimensions did not get appropriate attention and is not invariably satisfied. While the indentation is much smaller than the horizontal dimensions of the cell surface, the question that remains to be answered is whether it is also smaller than the sample thickness.

## Membrane Pressing in Between the Actomyosin Cortex Filaments

In some cases, the cell membrane can be pressed in between the actomyosin cortex filaments with negligible effect on other cell components. Then, in the context of the “half-space” assumption, the relevant length to be compared to the indentation is the thickness of the membrane rather than the width of the cell. Importantly, the membrane thickness is a few nanometers only—much smaller than normal indentation depths that are in the range of hundreds of nanometers. It is crucial to identify those cases since then the “half-space” assumption is not fulfilled and 2D models are needed.

**Figure 1:**
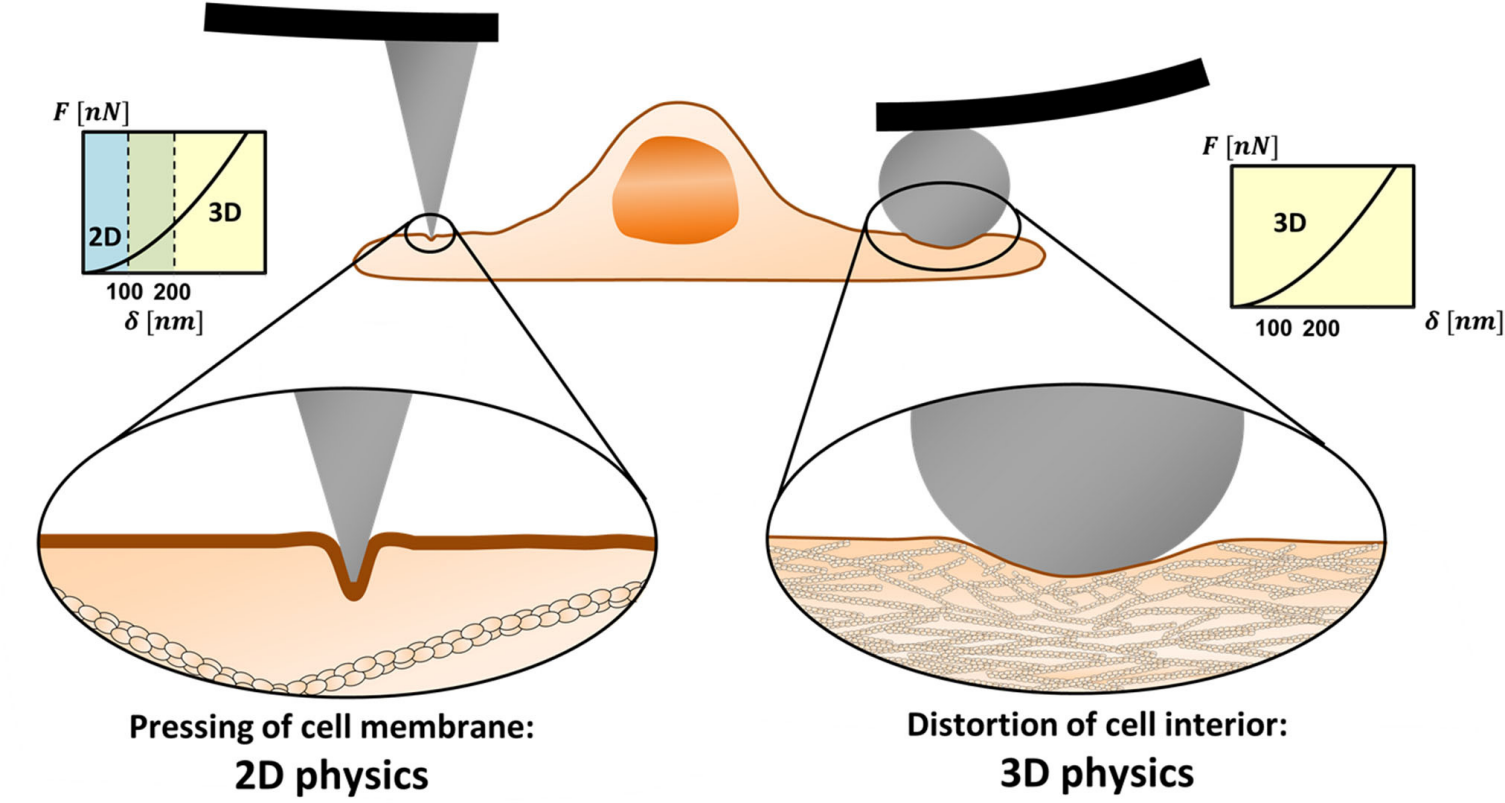
2D vs. 3D physics in mechanical measurements of cells using AFM. Sharp nanoprobes can squeeze the cell membrane in between the actomyosin cortex filaments (**left**). In low to medium range of indentation depths (typically ≤100 nm), other cell components may not be affected by the tip load. Then, the membrane resistance to the load needs to be represented by 2D physics rather than by 3D (contact mechanics) theories. When a microprobe is used, the load diameter is larger than the cortex mesh and the system obeys 3D physics even for relatively shallow indentations (**right**).

The ratio between the cortex mesh and the indentation diameter determines whether the AFM experiments are to be analyzed by 3D or 2D theories or by integrative models. Figure 1 demonstrates the length scales in two distinct scenarios. On the left side, the probe is a tip with a few-nanometer apex, and on the right, a colloidal microbead. In the latter case, the probe diameter is larger than the cortex mesh size causing a distortion of the cytoskeleton. In contrast, when a sharp tip is used, due to its small diameter, it often leads to protrusion of the membrane into the actin cytoskeleton. The load would affect inner cell component only at larger depth.

The cortex mesh size, typically 100–200 nm, differs between cell types, and within local regions of the cell (Frey, 2002; Eghiaian et al., 2015; Chugh and Paluch, 2018; Svitkina, 2020). The typical thickness of the cortex layer is in the scale of hundreds of nanometers (Svitkina, 2020). The indentation depth in cell experiments varies between tens to hundreds of nanometers and, in some cases, reaches micron scales, depending on the intracellular structure of interest (Gavara, 2017). Typical cones and pyramidal tips have an opening angle of 25° or less. Thus, indentation diameters on the order of 100 nm, which can be comparable to the cortex mesh size, may be achieved when tips of a-few-nanometer apexes are indented to depths larger than 100 nm. This roughly defines the conditions where the system obeys 2D rather than 3D physics.

## Insight From the 2D Physics Regime

Nanoprobe indentations present large heterogeneity and are very sensitive to the pressing location due to local cytoskeletal or polymer brush elements (Guz et al., 2014; Kilpatrick et al., 2015; Wu et al., 2018). Accordingly, *F*–δ plots vary in their shape; those that are not above the cell nucleus and have the smallest slope are most likely due to shallow membrane squeezing in between the cytoskeleton filaments. The approach parts of the curves represent the sample elasticity with negligible effects of the viscosity (Efremov et al., 2017). Thus, while these cases should not be represented by contact mechanics (or by other 3D theories), analyzing them with 2D models can provide information about the membrane elasticity, as outlined below.

The elastic energy of cell membranes is commonly described by Helfrich Hamiltonian (Helfrich and Jakobsson, 1990) with the leading terms given by $\frac{1}{2}\kappa_{bend}\int dA{\left(c_{1}+c_{2}\right)}^{2}+\frac{1}{2}\kappa_{stretch}\frac{{\left(A-A_{0}\right)}^{2}}{A_{0}}$. The first term describes the bending energy where κ_*bend*_ is the bending modulus, *c*_1_ and *c*_2_ are the membrane principal curvatures, and *dA* is a surface element. A Gaussian term is irrelevant here since there is no topological membrane remodeling and the spontaneous curvature is assumed to be zero (Kozlov, 2018). This integral normally depends linearly on the indentation depth due to the inverse proportion between the local surface area and the principal curvatures (Brill-Karniely et al., 2020). Therefore, the main contribution to the dependence of the force on δ comes from the second term. The latter represents the lowest order of the in-plane tension energy where κ_*stretch*_ is the membrane stretching modulus, and *A* and *A*_0_ are, respectively, the perturbed and unperturbed surface areas of the membrane. The leading term of the indentation force is thus $F=\kappa_{stretch}\frac{A-A_{0}}{A_{0}}$. Using this expression, we can obtain an intuitive description of membrane protrusion due to tip indentation. Similar to the basic Hertz model of contact mechanics, we ignore contributions of cell–probe attractive interactions. When the membrane is not adhered to the probe, it is basically pressed by the sharp edge of the tip with negligible effect of the tip geometry. Before the probe reaches inner cell components, the membrane is pressed in between the cortex filaments, acquiring an amorphous geometry of a misshaped cone. In the simple case of a cone, the force–indentation relation is given by

(2) $$\begin{array}{l} F=\kappa_{stretch}\sqrt{\frac{\delta^{2}}{x^{2}}+1} \end{array}$$

where *x* is the cone radius that can be approximated by half cortex mesh size. Interestingly, this simplified expression is analog to a power law with an exponent ranging between 1 and 2, similar to contact mechanics solutions (Equation 1). This may explain why AFM curves are fitted well with contact mechanics models also when the system should be described by 2D physics.

An important consequence of this discussion is that the membrane extension modulus can be directly estimated from fitting force indentation plots to a 2D model. This can be done by analyzing cell indentations with nanometer apex tips in a depth range of ~100 nm. Among the heterogenic *F*–δ curves of a given cell, those with the lowest slopes need to be chosen to avoid influence of direct load on cortex filaments. As an example, we can consider breast cancer cells (MCF-7) indented with sharp conical probes (Wu et al., 2018). These indentations, which show large heterogeneity, are usually analyzed with Sneddon theory; a contact mechanics-based model in which Equation 1 is quadratic (Kilpatrick et al., 2015; Wu et al., 2018). Using the contact mechanics power law fit, we can define the slope threshold. For the example of the breast cancer cells, this can be a prefactor of 0.015 mN/m in a quadratic fit of the force indentation curves for shallow (<100 nm) indentation range. Using *x* =100 nm in Eq. 2, one obtains a value of 0.35 N/m for κ_*stretch*_, similar to the values measured with other methods (Evans and Needham, 1987; Rawicz et al., 2000).

## Discussion

The validity of contact mechanics models in analyzing AFM force spectroscopy of cells has been previously addressed regarding effects like attractive interactions with the probe, special membrane geometries, and effective stiffening when approaching the underlying surface (Dimitriadis et al., 2002; Roa et al., 2011; Gavara and Chadwick, 2012; Guz et al., 2014; Kilpatrick et al., 2015). Here, an additional aspect is given with respect to membrane distortion and the system dimensionality. Previous theories that accounted for the membrane contribution in indentation experiments (for example, using finite element models) treated the cytoskeleton and other cell components as a homogeneous continuum (Hajji, 1978; Lulevich et al., 2010; Zhang and Zhang, 2011). However, when the load area is smaller than the cortex mesh, the indented membrane is floating on top of discrete anchors rather than placed on a uniform material. These indentations perturb the 2D membrane with negligible effect on the 3D cell bulk. Indentations with AFM probes of a-few-nanometer apexes may cause pressing of the membrane in between the cortex filaments for depths <100 nm. The load would start affecting inner cell components at deeper indentations, and in a fitting range larger than 200 nm, 3D theories such as contact mechanics would be appropriate. A more specific definition of the indentation depth at which there is a transition between optional 2D and 3D physics depends on the actomyosin cortex density of the tested cell type. Importantly, using 2D models (such as the simplified expression in Equation 2), membrane extension moduli can be obtained from shallow <100 nm indentations of sharp tips without pre-knowledge about the Young's modulus of the cell bulk. This can be done by analyzing force indentation plots that have low slopes, defined by a threshold.

## Author Contributions

YB-K conceived the work and wrote the paper.

## Conflict of Interest

The author declares that the research was conducted in the absence of any commercial or financial relationships that could be construed as a potential conflict of interest.
